# USP10 deubiquitylates and stabilizes DIRAS2 to suppress the growth of pancreatic cancer cells

**DOI:** 10.1002/mco2.751

**Published:** 2024-09-23

**Authors:** Qian Chen, Xiufang Xiong, Yi Sun

**Affiliations:** ^1^ Cancer Institute (Key Laboratory of Cancer Prevention and Intervention China National Ministry of Education) of the Second Affiliated Hospital and Institute of Translational Medicine Zhejiang University School of Medicine Hangzhou China; ^2^ Research Center for Life Science and Human Health of Binjiang Institute Zhejiang University Hangzhou China

Dear Editor,

The stability of a short‐lived protein is precisely regulated by its E3 ligase for degradation and its deubiquitylase (DUB) for stabilization, and the imbalanced activity between E3s and DUBs contributes to the development of some diseases, including cancer.^1^ The DIRAS family has three members, which are distinct branches of GTPases with anti‐RAS activity.^2^, ^3^ Our most recent study identified CRL5^ASB11^ as an E3 ligase for targeted ubiquitylation and degradation of DIRAS2. Biologically, inactivation of Crl5^Asb11^ caused Diras2 accumulation to suppress *Kras^G12D^*‐induced mouse pancreatic tumorigenesis by blocking the Ras‐Mapk‐cMyc signals.^4^ However, which DUB is responsible for DIRAS2 stabilization is previously unknown.

To this end, we generated PANC1 cells stably expressing FLAG‐tagged DIRAS2 (Figure S1A), followed by affinity purification and mass spectrometry analysis in an attempt to identify DIRAS2 binding proteins (Figure S1B). Among the 131 candidates (Table S1), USP10 was the only DUB identified. We then confirmed that DIRAS2 indeed selectively bound to USP10 among a total of 9 DUBs tested (Figure 1A, top). We further confirmed that endogenous DIRAS2 bound to endogenous USP10 in PANC1 pancreatic cancer cells, indicating that the binding is physiologically relevant (Figure 1A, bottom).

**FIGURE 1:**
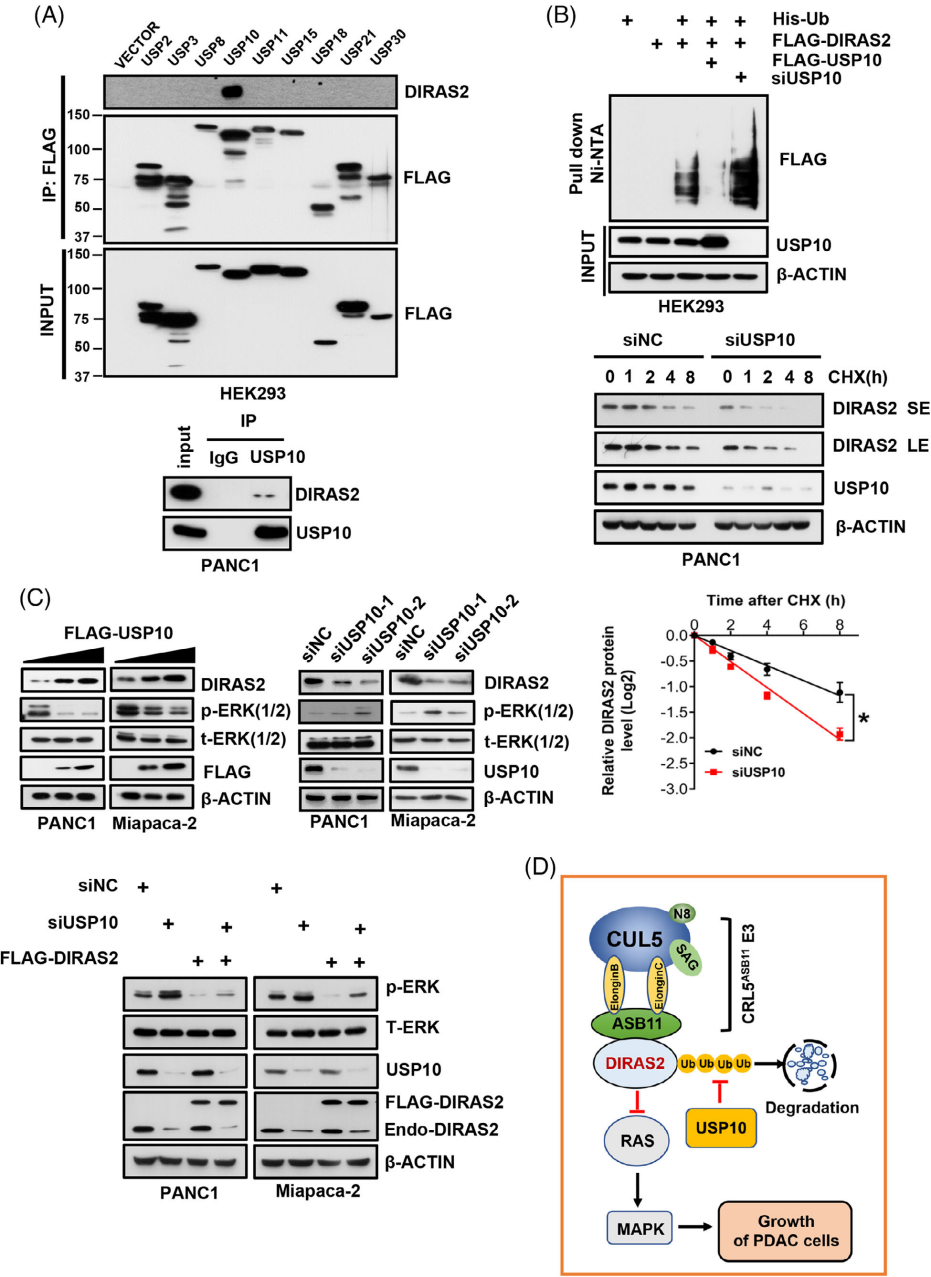
USP10 deubiquitinates and stabilizes DIRAS2 to suppress the growth of pancreatic cancer cells. (A) USP10 binds with DIRAS2. HEK293 cells were transfected with indicated FLAG‐tagged deubiquitylase (DUB) constructs for 48 h and then harvested for immunoprecipitation (IP) with FLAG beads, followed by immunoblotting (IB) analysis with indicated antibodies (Abs) (top). PANC1 cells were harvested for IP with USP10 antibody, along with normal immunoglobulin G (IgG) as a control, followed by IB analysis with indicated Abs (bottom). The 10% of whole cell extracts were used as the input. (B) USP10 regulates DIRAS2 polyubiquitylation and its protein half‐life. HEK293 cells were transfected with indicated constructs and siRNA oligos for 48 h and then harvested for Ni‐NTA purification after 6 h of MG132 treatment. The purified fractions and 10 % of whole cell extracts, as the input, were analyzed by IB analysis with indicated Abs (top). PANC1 cells were transfected with indicated siRNA oligos for 48 h and then treated with CHX (50 µg/ml) for indicated time periods before being harvested for IB analysis (middle) SE: short exposure; LE: longer exposure. Densitometry quantification was analyzed with ImageJ, and the half‐life curves are shown (bottom). Data shown are mean ± SEM (*n* = 3). * *p* < 0.05. (C) USP10 positively regulates DIRAS2 levels to inactivate the RAS‐MAPK signal. PANC1 and Miapaca‐2 cells were transfected with the increasing amount of FLAG‐USP10 construct (top left) or indicated siRNA oligos (top right) for 48 h, and then harvested for IB analysis. PANC1 and Miapaca‐2 cells were transfected with indicated siRNA oligos for 24 h, then transfected with FLAG‐DIRAS2 or mock vector (bottom) for 48 h, and then harvested for IB analysis. (D) A working model: USP10 or CRL5^ASB11^ E3 positively or negatively affects the DIRAS2 stability, respectively. USP10 knockdown causes DIRAS2 accumulation to inactivate the MAPK signal and inhibit the growth of PDAC cells.

We then characterized whether USP10 is the DUB for DIRAS2. Indeed, USP10 overexpression completely blocked DIRAS2 polyubiquitylation (Figure 1B, top), and extended its protein half‐life (Figure S1C), whereas USP10 knockdown significantly enhanced DIRAS2 polyubiquitylation (Figure 1B, top), and shortened its protein half‐life (Figure 1B, middle and bottom). Likewise, in both PANC1 and Miapaca‐2 pancreatic cancer cells, USP10 overexpression increased DIRAS2 levels and inactivated pERK1/2 in a dose‐dependent manner (Figure 1C, top left), whereas USP10 knockdown decreased DIRAS2 levels and activated pERK1/2 (Figure 1C, top right).

We finally performed an important rescue experiment and found that the activation of pERK1/2 by USP10 knockdown was completely abrogated by simultaneous ectopic expression of DIRAS2 at the endogenous levels (Figure 1C, bottom), firmly demonstrating that DIRAS2 plays a causal role in USP10 regulation of MAPK signals. Taken together, USP10 is a bona fide DUB that stabilizes DIRAS2 through deubiquitylation.

Biologically, given that DIRAS2 plays a tumor suppressor role in pancreatic ductal adenocarcinoma (PDAC) induced by *Kras^G12D^* ^4^, we hypothesized that USP10 would likely regulate the growth of pancreatic cancer cells via stabilizing DIRAS2. Indeed, USP10 knockdown promoted the growth of both PANC1 and Miapaca‐2 cells (Figure S1D, top). However, USP10 overexpression had no effect on the growth of pancreatic cancer cells (Figure S1D, bottom).

USP10 acts as either a tumor suppressor or oncogene in a manner dependent on the function of its substrates.^5^ Specifically, by deubiquitylating and stabilizing p53, USP10 inhibited growth of tumor cells harboring wild‐type p53.^6^ By deubiquitylating and stabilizing KLF4, USP10 suppressed *Kras^G12D^*‐induced lung tumorigenesis.^7^ Likewise, by binding and deubiquitylating Pten, USP10 inhibited the growth and invasion of lung cancer cells.^8^ On the other hand, USP10 deubiquitinated and stabilized YAP/TAZ to promote the proliferation of liver cancer cells,^9^ and by deubiquitinating PABPC1, USP10 increased CLK2 translation to promote the growth of pancreatic cancer cells.^10^ Thus, the net biological effects upon USP10 manipulations are likely to be context and cell‐line dependent. The full rescue of MAPK activation upon USP10 knockdown by DIRAS2 ectopic expression shown in this study indicates that the effect of USP10 on the MAPK signal is mediated by DIRAS2 in pancreatic cancer cells. Nevertheless, the future study should be directed to determine the in vivo role of *Usp10* in pancreatic tumorigenesis, induced by *Kras^G12D^*.

In summary, our study identified USP10 as a DUB for DIRAS2, which couples with CRL5^ASB11^ E3 to regulate DIRAS2 stability. USP10 knockdown promotes the growth of PDAC cells by enhancing DIRAS2 polyubiquitylation and shortening its protein half‐life, leading to activation of the RAS‐MAPK signal (Figure 1D).

## AUTHOR CONTRIBUTIONS

Qian Chen performed experiments; Qian Chen, Xiufang Xiong, and Yi Sun analyzed data; Qian Chen, Xiufang Xiong, and Yi Sun wrote the manuscript. Yi Sun finalized the manuscript. Yi Sun conceived and supervised the project. All authors have read and approved the final manuscript.

## CONFLICT OF INTEREST STATEMENT

The authors declare no conflict of interest.

## ETHICS STATEMENT

Not applicable

## Supporting information

Supporting Information

## Data Availability

The data are available from the corresponding author upon reasonable request.
