# Supplementary material for: USP10 deubiquitylates and stabilizes DIRAS2 to suppress the growth of pancreatic cancer cells
Source: MedComm (2020). 2024 Sep 23;5(10):e751. doi: 10.1002/mco2.751 (PMC11417426; doi:10.1002/mco2.751)
Supplement: Supplementary file 1 — Supporting Information [file MCO2-5-e751-s001.pdf]

USP10 deubiquitylates and stabilizes DIRAS2 to suppress the growth of pancreatic cancer cells

Qian Chen[1], Xiufang Xiong[1] and Yi Sun[1,2,*]

[1]Cancer Institute (Key Laboratory of Cancer Prevention and Intervention, China National Ministry of Education) of the Second Affiliated Hospital and Institute of Translational Medicine, Zhejiang University School of Medicine, Hangzhou 310029, China.

[2]Research Center for Life Science and Human Health of Binjiang Institute, Zhejiang University, Hangzhou 310053, China.

[*]To whom correspondence should be addressed: Yi Sun, yisun@zju.edu.cn

Chen, et al Figure S1

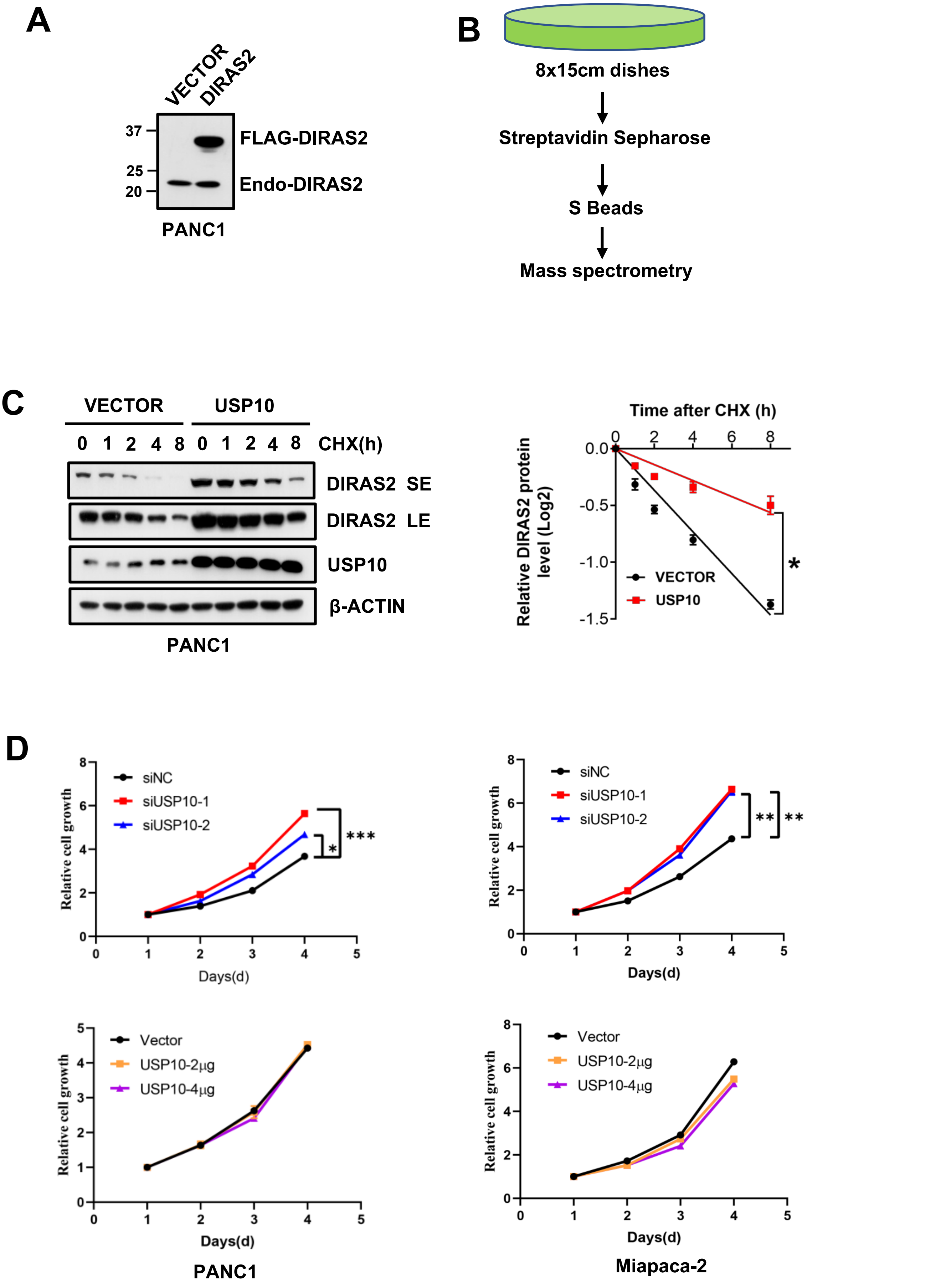

Legends for supplementary figure

Figure S1. USP10 deubiquitylates and stabilizes DIRAS2 to suppress the growth of pancreatic cancer cells.

A: Validation of DIRAS2 overexpression in PANC1 cells stably expressing FLAG-tagged DIRAS2. PANC1 cells were transfected with mock vector or FLAG-DIRAS2 for 48 h and then selected for stable expression with G418, followed by immunoblotting (IB) analysis.

B: Schematic diagram of identification of DIRAS2 binding proteins. PANC1 cells stably expressing FLAG-tagged DIRAS2 or a mock vector were harvested and subjected to two rounds of purification using Streptavidin Sepharose and S beads. The eluted proteins were analyzed by mass spectrometry for their identities.

C: Overexpression of USP10 extends the protein half-life of DIRAS2. PANC1 cells were transfected with indicated constructs for 48 h and then treated with CHX (50μg/ml) for indicated time periods before being harvested for IB analysis (left). Densitometry quantification was analyzed with ImageJ, and the half-life curves are shown (right). Data shown are mean ± SEM of three independent experiments. *$p < 0.05$. SE: shorter exposure. LE: longer exposure.

D: USP10 regulates the growth of PDAC cells. PANC1 (left) and Miapaca-2 (right) cells were transfected with indicated siRNA oligos (top) or constructs (bottom) for 48 h and then subjected to CCK8 growth assay. Data shown are mean ± SEM of three independent experiments. ns, not significant; * $p < 0.05$, ** $p < 0.01$, *** $p < 0.001$.

Material and methods

Cell culture and generation of PANC1 cells stably expressing DIRAS2

Human embryonic kidney HEK293 cells and pancreatic cancer PANC1 and Miapaca-2 cells were maintained in Dulbecco's Modified Eagle's Medium (DMEM) containing 10% (v/v) fetal bovine serum (FBS) and 1% penicillin-streptomycin. PANC1 cells were transfected with pIRES2-FLAG-DIRAS2 or mock vector for 48 h, and then selected with G418 (Merck, Cat.108321-42-2) for 2 weeks.

Affinity purification

The affinity purification was performed as described.[1] Briefly, PANC1 cells stably expressing FLAG-DIRAS2 or mock vector were harvested and lysed with NETN buffer (20 mM Tris-HCl [pH 8.0], 100 mM NaCl, 1 mM EDTA, 0.5% Nonidet P-40) containing inhibitors of protease and phosphatase. The cell lysates were incubated with Streptavidin Sepharose (Thermo Fisher, Cat.20359) for 1 h at 4 °C. After washing, the bound proteins were eluted in 1 mg/ml Biotin for 30 min at 4 °C, and then incubated with S beads (Millipore, Cat.3800262) for 1 h at 4 °C. Finally, the DIRAS2 binding proteins were eluted by boiling in SDS sample buffer for 10 min, and then resolved in the SDS-PAGE gels. The sample-containing gels were cut after a short run with all the samples entering the running gels, and submitted to PTMbio (Hangzhou, China) for mass spectrum detection.

Transfection

Cells were transfected with plasmids or siRNA oligos (synthesized by Ribobio, Hangzhou, China), using Lipofectamine 3000 (Invitrogen) according to the manufacturer's instructions. The sequences of siRNA are as follow: siNC: 5′-AUU GUA UGC GAU CGC AGA C-3′, siUSP10-1: 5′-CCC TGA TGG TAT CAC TAA A-3′, siUSP10-2: 5′-CCA AGG TTA TAC CAC AAA A-3′.

In vivo ubiquitylation assay

The *in vivo* ubiquitylation assay was performed as described.[2] Briefly, cell pellets were lysed in lysis buffer (6 M guanidinium-HCl, 0.1 M Na_2HPO4/NaH_2PO4, 10 mM Tris–HCl (pH 8.0), 10 mM β-mercaptoethanol) and incubated with Ni-NTA beads (Qiagen, Cat.No:30210) at room temperature for 4 h. After beads washing, the proteins were eluted from beads with imidazole and then analyzed by Western blotting.

Reference:

1 Chang, Y. *et al.* The UBE2F-CRL5(ASB11)-DIRAS2 axis is an oncogene and tumor suppressor cascade in pancreatic cancer cells. *Dev Cell* **59**, 1317-1332.e1315, doi:10.1016/j.devcel.2024.03.018 (2024).

2 Zhao, Y., Xiong, X. & Sun, Y. DEPTOR, an mTOR inhibitor, is a physiological substrate of SCF(βTrCP) E3 ubiquitin ligase and regulates survival and autophagy. *Mol Cell* **44**, 304-316, doi:10.1016/j.molcel.2011.08.029 (2011).

Table S1. A list of DIRAS2 binding proteins

Accession	Protein names	Gene names	MW [kDa]	Protein score	Sequence coverage (%)	# Unique Peptides	# Peptides	# PSMs	Abundances
Q15149	Plectin	PLEC	531.5	6352	37	140	140	173	2.57E+09
P48681	Nestin	NES	177.3	988	22	26	26	28	1.48E+08
Q15233	Non-POU domain-containing octamer-binding protein	NONO	54.2	798	42	13	13	24	2.48E+08
P52306	Rap1 GTPase-GDP dissociation stimulator 1	RAP1GDS1	66.3	739	32	14	14	19	5.18E+08
Q9NZI8	Insulin-like growth factor 2 mRNA-binding protein 1	IGF2BP1	63.4	601	27	10	12	16	1.48E+08
O00425	Insulin-like growth factor 2 mRNA-binding protein 3	IGF2BP3	63.7	556	20	6	8	12	4.39E+07
Q13283	Ras GTPase-activating protein-binding protein 1	G3BP1	52.1	540	31	9	12	15	1.29E+08
P51114	Fragile X mental retardation syndrome-related protein 1	FXR1	69.7	534	30	13	13	15	1.03E+08
Q9Y6M1	Insulin-like growth factor 2 mRNA-binding protein 2	IGF2BP2	66.1	530	22	8	9	12	5.24E+07
P11940	Polyadenylate-binding protein 1	PABPC1	70.6	467	18	7	9	14	2.13E+08
P68371	Tubulin beta-4B chain	TUBB4B	49.8	367	20	1	8	10	8.41E+06
Q13310	Polyadenylate-binding protein 4	PABPC4	70.7	362	18	7	9	12	1.26E+08
Q9UN86	Ras GTPase-activating protein-binding protein 2	G3BP2	54.1	313	15	3	6	9	1.90E+07
Q08211	ATP-dependent RNA helicase A	DHX9	140.9	312	9	9	9	9	2.26E+07
P09651	Heterogeneous nuclear ribonucleoprotein A1	HNRNPA1	38.7	302	25	5	6	8	2.79E+07
Q96HU8	GTP-binding protein Di-Ras2	DIRAS2	22.5	272	37	5	5	7	3.04E+08
Q7Z417	Nuclear fragile X mental retardation-interacting protein 2	NUFIP2	76.1	267	18	6	6	7	1.47E+07
P26599	Polypyrimidine tract-binding protein 1	PTBP1	57.2	267	14	4	4	5	3.73E+07
Q8WXF1	Paraspeckle component 1	PSPC1	58.7	255	10	3	3	4	2.39E+07
Q92900	Regulator of nonsense transcripts 1	UPF1	124.3	249	12	9	9	10	1.71E+07
P27694	Replication protein A 70 kDa DNA-binding subunit	RPA1	68.1	225	13	4	4	5	3.84E+06
Q06787	Synaptic functional regulator FMR1	FMR1	71.1	220	9	5	5	5	2.16E+07
O60506	Heterogeneous nuclear ribonucleoprotein Q	SYNCRIP	69.6	218	19	8	8	8	2.63E+07
Q6PKG0	La-related protein 1	LARP1	123.4	203	8	5	5	5	1.58E+07
Q04637	Eukaryotic translation initiation factor 4 gamma 1	EIF4G1	175.4	197	5	6	6	6	1.22E+07
P26196	Probable ATP-dependent RNA helicase DDX6	DDX6	54.4	190	18	6	6	7	1.19E+07
P22626	Heterogeneous nuclear ribonucleoproteins A2/B1	HNRNPA2B1	37.4	186	16	4	5	5	1.12E+07
Q12905	Interleukin enhancer-binding factor 2	ILF2	43	169	11	3	3	3	8.89E+06
P52597	Heterogeneous nuclear ribonucleoprotein F	HNRNPF	45.6	165	14	2	4	5	1.56E+06
P35579	Myosin-9	MYH9	226.4	163	3	5	5	5	9.01E+06
Q9Y2T7	Y-box-binding protein 2	YBX2	38.5	150	18	3	4	5	7.33E+06
P51116	Fragile X mental retardation syndrome-related protein 2	FXR2	74.2	143	6	3	3	3	6.78E+06
Q9Y224	RNA transcription, translation and transport factor protein	RTRAF	28.1	133	15	2	2	2	2.19E+06
P39060	Collagen alpha-1(XVIII) chain	COL18A1	178.1	133	2	2	2	2	2.69E+06
P17844	Probable ATP-dependent RNA helicase DDX5	DDX5	69.1	129	6	2	3	3	1.01E+07
O15061	Synemin	SYNM	172.7	126	4	4	4	4	5.99E+06
Q9Y520	Protein PRRC2C	PRRC2C	316.7	125	3	7	7	7	1.88E+07
Q8NCA5	Protein FAM98A	FAM98A	55.2	124	8	2	2	2	4.83E+06
Q14257	Reticulocalbin-2	RCN2	36.9	123	17	3	3	3	7.15E+06
P39019	40S ribosomal protein S19	RPS19	16.1	119	35	5	5	5	1.61E+08
Q92499	ATP-dependent RNA helicase DDX1	DDX1	82.4	116	6	3	3	4	6.63E+06
B5ME19	Eukaryotic translation initiation factor 3 subunit C-like protein	EIF3CL	105.4	114	4	3	3	3	4.17E+06
O00303	Eukaryotic translation initiation factor 3 subunit F	EIF3F	37.5	113	9	2	2	2	2.84E+06
Q14694	Ubiquitin carboxyl-terminal hydrolase 10	USP10	87.1	111	8	4	4	5	1.48E+07
P16989	Y-box-binding protein 3	YBX3	40.1	109	10	1	2	2	
Q13813	Spectrin alpha chain, non-erythrocytic 1	SPTAN1	284.4	108	2	3	3	3	6.38E+05
P0DP24	Calmodulin-2	CALM2	16.8	104	30	4	4	4	1.92E+07
Q92841	Probable ATP-dependent RNA helicase DDX17	DDX17	80.2	97	6	2	3	3	2.47E+07
P51991	Heterogeneous nuclear ribonucleoprotein A3	HNRNPA3	39.6	93	8	2	2	2	3.37E+06
O00159	Unconventional myosin-Ic	MYO1C	121.6	93	5	4	4	4	6.88E+06
P50402	Emerin	EMD	29	91	13	2	2	2	4.78E+06
O43795	Unconventional myosin-Ib	MYO1B	131.9	88	4	3	3	3	2.66E+06
Q9Y262	Eukaryotic translation initiation factor 3 subunit L	EIF3L	66.7	88	5	2	2	2	1.13E+06
Q14444	Caprin-1	CAPRIN1	78.3	87	6	3	3	3	2.65E+07
Q96EP5	DAZ-associated protein 1	DAZAP1	43.4	84	4	1	1	1	3.97E+06
O15372	Eukaryotic translation initiation factor 3 subunit H	EIF3H	39.9	83	5	1	1	1	
Q15717	ELAV-like protein 1	ELAVL1	36.1	80	15	4	4	4	1.32E+07
P25398	40S ribosomal protein S12	RPS12	14.5	70	23	2	2	2	4.43E+06
P43243	Matrin-3	MATR3	94.6	66	6	3	3	3	1.08E+07
Q01082	Spectrin beta chain, non-erythrocytic 1	SPTBN1	274.4	65	1	2	2	2	3.01E+06
Q5PRF9	Protein Smaug homolog 2	SAMD4B	75.4	64	2	1	1	1	3.88E+05
P12956	X-ray repair cross-complementing protein 6	XRCC6	69.8	62	6	2	2	2	1.33E+06
P62081	40S ribosomal protein S7	RPS7	22.1	60	15	2	2	2	
P36578	60S ribosomal protein L4	RPL4	47.7	57	3	1	1	1	2.49E+06
P62753	40S ribosomal protein S6	RPS6	28.7	55	5	1	1	1	3.15E+06
P12814	Alpha-actinin-1	ACTN1	103	54	1	1	1	1	2.16E+06
Q99700	Ataxin-2	ATXN2	140.2	53	1	1	1	1	
P62244	40S ribosomal protein S15a	RPS15A	14.8	53	11	1	1	1	
P41091	Eukaryotic translation initiation factor 2 subunit 3	EIF2S3	51.1	51	3	1	1	1	7.55E+05
Q96I24	Far upstream element-binding protein 3	FUBP3	61.6	50	2	1	1	1	2.37E+06
P63010	AP-2 complex subunit beta	AP2B1	104.5	49	2	1	1	1	1.10E+06
P05388	60S acidic ribosomal protein P0	RPLP0	34.3	49	7	2	2	2	5.52E+06
P15144	Aminopeptidase N	ANPEP	109.5	47	1	1	1	1	1.06E+06
P23396	40S ribosomal protein S3	RPS3	26.7	46	5	1	1	1	3.61E+06
Q71UM5	40S ribosomal protein S27-like	RPS27L	9.5	46	15	1	1	1	2.33E+06
P61254	60S ribosomal protein L26	RPL26	17.2	45	6	1	1	1	5.19E+06
Q9Y3I0	RNA-splicing ligase RtcB homolog	RTCB	55.2	45	6	2	2	2	6.16E+06
P07900	Heat shock protein HSP 90-alpha	HSP90AA1	84.6	45	3	2	2	2	2.18E+07
Q9NZB2	Constitutive coactivator of PPAR-gamma-like protein 1	FAM120A	121.8	44	2	1	1	1	
P42766	60S ribosomal protein L35	RPL35	14.5	44	15	2	2	2	1.14E+07
P62891	60S ribosomal protein L39	RPL39	6.4	40	20	1	1	1	2.51E+06
O75821	Eukaryotic translation initiation factor 3 subunit G	EIF3G	35.6	40	5	1	1	1	1.25E+06
Q00610	Clathrin heavy chain 1	CLTC	191.5	40	1	1	1	1	8.87E+05
O75531	Barrier-to-autointegration factor	BANF1	10.1	39	27	1	1	1	2.50E+06
P62899	60S ribosomal protein L31	RPL31	14.5	39	11	1	1	1	7.33E+06
P18621	60S ribosomal protein L17	RPL17	21.4	39	5	1	1	1	3.10E+06
P21333	Filamin-A	FLNA	280.6	38	0	1	1	1	8.30E+05
Q9H0D6	5'-3' exoribonuclease 2	XRN2	108.5	38	2	1	1	1	5.86E+05
P84090	Enhancer of rudimentary homolog	ERH	12.3	38	11	1	1	1	6.16E+06
P63173	60S ribosomal protein L38	RPL38	8.2	37	19	1	1	1	3.62E+06
O43809	Cleavage and polyadenylation specificity factor subunit 5	NUDT21	26.2	37	7	1	1	1	8.44E+05
P15880	40S ribosomal protein S2	RPS2	31.3	37	9	2	2	2	8.07E+06
Q07955	Serine/arginine-rich splicing factor 1	SRSF1	27.7	37	6	1	1	1	1.25E+06
P60660	Myosin light polypeptide 6	MYL6	16.9	36	6	1	1	1	2.83E+06
Q96AG4	Leucine-rich repeat-containing protein 59	LRRC59	34.9	35	4	1	1	1	
Q8IVF7	Formin-like protein 3	FMNL3	117.1	35	1	1	1	1	8.97E+05
P46781	40S ribosomal protein S9	RPS9	22.6	34	8	1	1	1	8.98E+05
P62987	Ubiquitin-60S ribosomal protein L40	UBA52	14.7	34	7	1	1	1	5.41E+06
Q96QR8	Transcriptional activator protein Pur-beta	PURB	33.2	32	10	1	1	1	1.28E+06
Q96S97	Myeloid-associated differentiation marker	MYADM	35.3	32	3	1	1	1	1.46E+06
O95816	BAG family molecular chaperone regulator 2	BAG2	23.8	32	4	1	1	1	2.12E+06
P60842	Eukaryotic initiation factor 4A-I	EIF4A1	46.1	31	4	1	1	1	
P61247	40S ribosomal protein S3a	RPS3A	29.9	31	5	1	1	1	2.33E+06
Q09666	Neuroblast differentiation-associated protein AHNAK	AHNAK	628.7	31	1	1	1	1	
P26368	Splicing factor U2AF 65 kDa subunit	U2AF2	53.5	31	2	1	1	1	1.77E+06
P60866	40S ribosomal protein S20	RPS20	13.4	30	9	1	1	1	2.20E+06
Q01130	Serine/arginine-rich splicing factor 2	SRSF2	25.5	30	7	1	1	1	1.05E+06
P62917	60S ribosomal protein L8	RPL8	28	30	4	1	1	1	2.22E+06
P62273	40S ribosomal protein S29	RPS29	6.7	30	20	1	1	1	4.18E+06
Q07020	60S ribosomal protein L18	RPL18	21.6	29	5	1	1	1	8.99E+05
O94973	AP-2 complex subunit alpha-2	AP2A2	103.9	29	3	1	1	1	4.87E+05
P06703	Protein S100-A6	S100A6	10.2	29	9	1	1	1	4.81E+06
P84243	Histone H3.3	H3-3A	15.3	29	5	1	1	1	3.14E+06
P05386	60S acidic ribosomal protein P1	RPLP1	11.5	29	14	1	1	1	2.88E+06
P35637	RNA-binding protein FUS	FUS	53.4	28	5	1	1	2	6.99E+06
Q9NUQ6	SPATS2-like protein	SPATS2L	61.7	28	2	1	1	1	
Q3MHD2	Protein LSM12 homolog	LSM12	21.7	28	5	1	1	1	2.96E+06
Q03113	Guanine nucleotide-binding protein subunit alpha-12	GNA12	44.3	28	3	1	1	1	3.11E+06
P80297	Metallothionein-1X	MT1X	6.1	27	13	1	1	1	8.68E+05
Q8N1A0	Keratin-like protein KRT222	KRT222	34.1	27	4	1	1	1	4.48E+06
P49327	Fatty acid synthase	FASN	273.3	26	0	1	1	1	5.38E+05
Q9HCE1	Helicase MOV-10	MOV10	113.6	26	1	1	1	1	2.55E+06
Q14573	Inositol 1,4,5-trisphosphate receptor type 3	ITPR3	303.9	26	0	1	1	1	2.24E+06
P62854	40S ribosomal protein S26	RPS26	13	25	6	1	1	1	1.30E+06
Q15517	Corneodesmosin	CDSN	51.5	25	3	1	1	1	2.09E+06
Q16891	MICOS complex subunit MIC60	IMMT	83.6	24	2	1	1	1	2.59E+06
O95793	Double-stranded RNA-binding protein Staufen homolog 1	STAU1	63.1	24	1	1	1	1	2.23E+06
O60437	Periplakin	PPL	204.6	24	1	1	1	1	8.76E+05
P14866	Heterogeneous nuclear ribonucleoprotein L	HNRNPL	64.1	24	2	1	1	1	1.25E+06
Q6ZRV2	Protein FAM83H	FAM83H	127	23	1	1	1	1	1.05E+06
P15927	Replication protein A 32 kDa subunit	RPA2	29.2	21	4	1	1	1	2.91E+06
